# Pharmacokinetic/pharmacodynamic analysis of linezolid in critically ill patients with severe sepsis treated with renal replacement therapy

**DOI:** 10.1186/2197-425X-3-S1-A880

**Published:** 2015-10-01

**Authors:** H Barrasa González, A Martín López, A Isla Ruiz, A Rodríguez Gascón, A Soraluce Olañeta, JA Sánchez Izquierdo, F Muñoyerro González, A Rodríguez Oviedo, S Castaño Ávila, F Fonseca San Miguel, FJ Maynar Moliner

**Affiliations:** Osakidetza, Vitoria, Spain; University of Basque Country, Vitoria, Spain; Doce de Octubre Hospital, Madrid, Spain; Joan XXIII Hospital, Tarragona, Spain

## Introduction

Pharmacokinetic (PK) of drugs in critically ill patients could vary from the general population. The pharmacokinetics of linezolid presents a high variability. Patients undergoing renal replacement therapy (RRT) could present lower linezolid concentration than expected. The pharmacokinetic/pharmacodynamic analysis (PK/ PD) is a useful tool to optimize dosing regimens of antibiotic therapy.

## Objectives

To analyse the PK/PD profile of linezolid (LZ) in patients with severe sepsis (SS) and RRT and to evaluate the efficacy of linezolid for the treatment of infections caused by gram-positive organisms.

## Methods

Study developed in three tertiary hospitals in patients with SS, RRT and treatment with LZ (600 mg q12 h). 8 each patient blood (prefilter and postfilter) and ultrafiltrate samples were taken at the following times: predose, 0,5, 1, 2, 3, 6, 8-10 and 12 h. Concentrations of LZ were determined by HPLC-UV. Demographic and laboratory data were collected, considering impaired liver function (ILF) as the elevation >2 times transaminase and/or elevated bilirubin and severe renal dysfunction (SRD) as the presence of CrCl < 15 ml/min. The PK study was performed using WinNonlin program. Calculation of the probability of successful treatment (PST) for minimum inhibitory concentrations (MIC) of 2, defined as the probability that the ratio area under the curve (AUC)/MIC is> 100 was performed. Quantitative variables were expressed as mean and standard deviation (SD), qualitative as percentages and were compared using the Student t test and Chi square test respectively. α significance level of 0,05.

## Results

26 patients (73 % male) were included. The mean age was 63 years (SD 15), with an average weight of 78 kg (SD 13). The mean dose of hemodiafiltration was 2,7 L/h (SD 0,7). The PK parameters were: mean plasma clearance (CL) of 8,9 L/h (SD 5,1), volume of distribution of 38,5 L (SD 10,1), elimination half-life of 3,8 h (SD 1,7) and AUC_0-24 h_ of 176,2 mg * h/L (SD 84,4). Patient with SRD and ILF had higher AUC (246 vs 139), higher C_min_ (4,6 vs 1,9) and less CL (5,4 vs 10,7), p < 0,02. The contribution of extracorporeal CL to the total CL was relevant in the presence of SRD (44% vs 20%, p < 0.01). The PK/PD target was only achieved in 38.4% of patients, 80% with SRD and ILF. The following factors were identified as predictors of treatment success: the presence of SRD [RR 4,8 (1,7-13,9)], SRD and ILF [RR 12,8 (1,9-87,6)], C_min_ >2 mg/L [RR 2,3 (1,3-4)], and the presence of a significant extracorporeal clearance (> 25%) [RR 5,3 (1,9-14,8)], p < 0,01.

## Conclusions

In our series, the current dose of linezolid allows achieving the PK/PD target in patients with SRD and ILF. In the absence of both dysfunctions, this aspect cannot be guaranteed. Although extracorporeal CL is significant in the presence of SRD, this impresses not be a limiting factor in achieving the PK/PD target.

## Grant Acknowledgment

Pfizer sponsored this study.

